# An alternative to skin graft for superficial surgical defect in oral cancer surgery

**DOI:** 10.1016/j.bjorl.2020.02.002

**Published:** 2020-03-11

**Authors:** Giancarlo Tirelli, Margherita Tofanelli, Alice Piccinato, Francesca Boscolo Nata

**Affiliations:** Azienda Sanitaria Universitaria Integrata di Trieste, Head and Neck Department, ENT Clinic, Trieste, Italy

**Keywords:** Bovine pericardium, Skin graft, Tutopatch, Oral cancer, Surgical defect

## Abstract

**Introduction:**

After surgery for oral cavity cancer, superficial surgical defects are usually covered with a skin graft that can be harvested with different thicknesses depending on the reconstructive need. Despite its popularity and efficacy, this solution has the disadvantage of excessive harvesting times and scarring of the donor site. Other surgeons have proposed the use of bovine pericardium as a reconstructive solution. Its use in otorhinolaryngology especially after oral cavity surgery has never been reported.

**Objective:**

The aim of this manuscript is to present our preliminary experience with the use of a collagen membrane obtained from bovine pericardium in the reconstruction of small and superficial defects after transoral resection of oral cavity tumors.

**Methods:**

A bovine collagen membrane was used to cover surgical defects in 19 consecutive patients undergoing transoral resection of small/superficial oral cancers. Photographs were obtained in the postoperative period to follow the healing process. We analyzed the pro and cons of this tool, recorded data on postoperative chewing-, speech- and taste-related quality of life, and tested the most appropriate settings providing the best reconstructive result.

**Results:**

The bovine collagen membrane allowed us to cover surgical defects of varying size in different oral sites. Shaping and placement proved to be simple. The membrane facilitated physiologic tissue repair: after one month it was completely absorbed and replaced by the patient’s own mucosa. No adverse features were observed in the cohort.

**Conclusion:**

A bovine collagen membrane can represent a fast and easy solution in cases of split-thickness defect. Unlike a skin graft, it is not associated with donor site morbidity and allows the patient’s own mucosa to be restored with a more physiological result.

## Introduction

The objective of oral cavity reconstruction after tumor resection is to restore the mucosal lining and cover the exposed bone, if any, to obtain both a functional and aesthetic result. Depending on the location and width and thickness of the defect, free and locoregional flaps or skin grafts may be used.^1^

Tutopatch® (Tutogen Medical GmbH, Germany) is a collagen membrane obtained from bovine pericardium that has undergone solvent dehydration and sterilization with gamma rays. It has been used in different surgical reconstructions, but at the time of this writing its use in ENT surgery is limited to the repair of the tympanic membrane perforation.^2^, ^3^ According to the manufacturer’s instructions, Tutopatch® facilitates closure of the tissue defect by acting as a template for the repair mechanism: over weeks or months, depending on the characteristics of the surgical defect and the patient’s comorbidities, the graft is progressively absorbed and replaced by the patient’s autologous tissue.

This article describes our experience with the use of Tutopatch® in transoral reconstruction after resection of small/superficial oral cavity cancers, as well as to cover the palatal bone.

## Methods

This prospective study was conducted in accordance with the Declaration of Helsinki and approved by our University Ethics Committee (nº 97/2019). Patients with moderate- or high-grade oral dysplasia or clinical T1‒2 oral cancer (according to the 8th edition of the TNM staging system) scheduled for transoral resection were enrolled in the study. Patients with locally advanced oral cancers who are better treated with expanded approaches followed by free-flap reconstruction were excluded.

The superficial resection margins were defined with Narrow Band Imaging (NBI)^4^ and then the tumor was resected by the same experienced surgeon in all the patients, using wave guide CO2 laser to result in the least lateral thermal damage^5^: lateral neck dissection was performed when indicated, in accordance with the National Comprehensive Cancer Network (NCCN) guidelines.^6^ After tumor resection and confirmation of frozen section negativity,^7^ we verified that the surgical defect did not need a bulky flap, that the surgical bed was regular and had a good blood supply, and that no communication had been created between the oral cavity and neck. If these conditions were met, we used a Tutopatch® collagen membrane to cover the surgical defect. This step could be performed by different members of our surgical team.

The Tutopatch® membrane was positioned over the surgical defect to obtain an impression of the size of the defect and shape the graft accordingly. Tutopatch® was then cut, keeping a margin of 2 mm beyond the impression so that the membrane edges exceeded beyond the mucosal defect margins, and it was placed with the rough surface facing the surgical defect.

**Figure 1:**
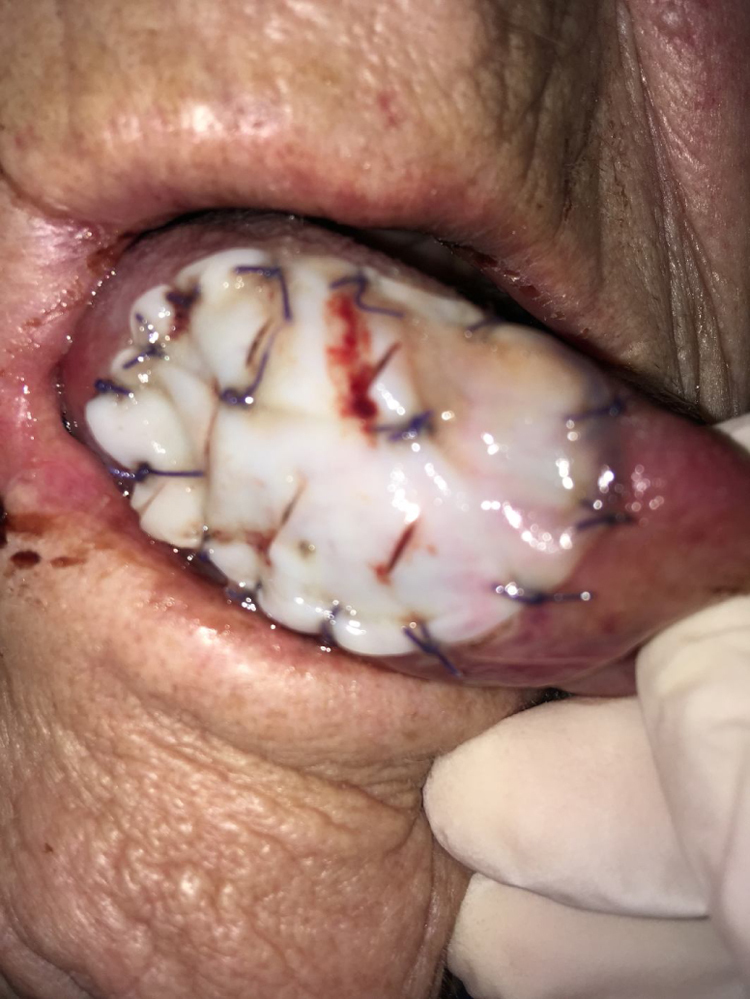
Tutopatch® is used to cover the surgical defect after resection of a pT1 of the right tongue. Absorbable stitches are used to fix the outer margins of Tutopatch® and two more absorbable stitches are placed to make the central part of the graft adhere to the underlying tissue and prevent graft lifting.

In the mobile tongue, floor of the mouth and cheek, absorbable stitches were used to fix the outer margins of Tutopatch®, and two more absorbable stitches were placed to make the central part of the graft adhere to the underlying tissue and prevent graft lifting (Fig. 1); alternatively, compression can be obtained by placing a thick gauze roll over the Tutopatch® for 4 days, as used in skin grafts. Conversely, in the hard palate, after periosteum preservation, only fibrin glue was used over the membrane. The anesthesiologist was informed of the reconstructive technique to prevent Tutopatch® aspiration during the pre-extubation maneuvers. The same information was given to the ENT department staff to prevent damage to the graft during the daily assessments.

As usually done in our Oral Surgery Department, a nasogastric tube (NGT) was placed at the end of the procedure to prevent food coming into contact with the surgical defect. Antibiotic therapy was prescribed for one week to prevent infection due to the contact between the wound and saliva. When returned to an oral diet, patients were instructed to do an antiseptic mouthwash after meals and to use hyaluronic acid spray to avoid biofilm contamination.^8^

During the hospitalization period, postoperative pain was checked daily using the numerical rating scale (NRS from 0 to 10)^9^ and the type and amount of pain killers were recorded. Photographs were taken daily during the hospital stay and then weekly for one month; thereafter the reconstructive result was observed during the monthly oncologic follow-up visits. One month after surgery patients were asked to complete the University of Washington Quality of Life Questionnaire: we specifically focused on chewing, speech and taste items (score from 0 worst to 100 best).^10^

## Results

A total of 19 patients were included in the study; 7 patients underwent concomitant neck dissection. Patient and tumor characteristics are reported in Table 1.

**Table 1:** Patient and tumor characteristics.

Variable	Results
Sex	
Male	10
Female	9
Age (y), mean ± SD (range)	68 ± 9 (55–83)
pT	
High grade dysplasia	3
T1	10
T2	6
Tumour location	
Hard palate	1
Cheek	6
Floor of the mouth	6
Tongue margin	6
Lateral neck dissection (nº of patients)	7
Monolateral	5
Bilateral	2
pN	
N0	5
N1	2
Feeding tube, days, mean ± SD (range)	6 ± 2 (3–10)

SD, Standard Deviation; pT, Pathologic tumor staging according the TNM 8th edition; pN, Pathologic node staging according the TNM 8th edition.

Grafts of different sizes (20 × 30 mm, 30 × 40 mm or 40 × 50 mm) were used according to the dimension of the surgical defect. In four patients with tumor of the floor of the mouth and ventral surface of the tongue the latter was sutured on itself to obtain a surgical defect dimension that could be easily covered with the graft.

Tutopatch® shaping and its placement proved to be simple, as demonstrated by the fact that also surgeons of less experience easily performed the procedure.

A nasogastric feeding tube was placed in the operating room in all patients but one, who refused and underwent parenteral feeding for 72 h: after a mean of 6 days (range 3–10 days) oral feeding was started with a soft cold diet and later patients returned to their usual diet at home. Patients reported mild postoperative pain (NRS 0–3) relieved by paracetamol 1 g three times a day maximum.

We observed complete healing of the surgical defect in all the patients. In the days following the reconstruction the graft was progressively re-absorbed and it was no longer visible 1 month after the procedure. One month postoperatively the mean reported scores for chewing; speech and taste were 64%, 73% and 83% respectively.

No patient complained about a foreign body sensation. There were no cases of wound dehiscence, infection, or adverse reaction.

## Discussion

The goal of oral cavity reconstruction is not only to cover the defect but also to restore function. Surgeons can choose among different reconstructive options of increasing complexity, and the choice should also take into account the patients’ comorbidities.^11^

Tutopatch® is a xenograft obtained from processed bovine pericardium and it is available in three different sizes.

Its mechanical characteristics have allowed its effective use in cardiac, digestive and ENT surgery, ophthalmology and neurosurgery. Specifically, in ENT surgery its use has been reported only for transcanal myringoplasty to reduce surgical times and the trauma related to graft harvesting.^2^

In our Department we used Tutopatch® to repair the surgical defect after oral dysplasia and early cancer resection, and this paper presents our preliminary results in 19 patients. In our experience Tutopatch® allowed efficient healing and closure of the surgical defect: there were no complications or cases of graft detachment regardless of the type of surgical bed (muscle or bone). As a technical note, we observed that if the graft is too wet its placement can become difficult because it tends to distort. Consequently, in contrast to the producer’s indications, we prefer not to rehydrate the graft with saline because it is efficiently moistened by the blood of the surgical defect.

**Figure 2:**
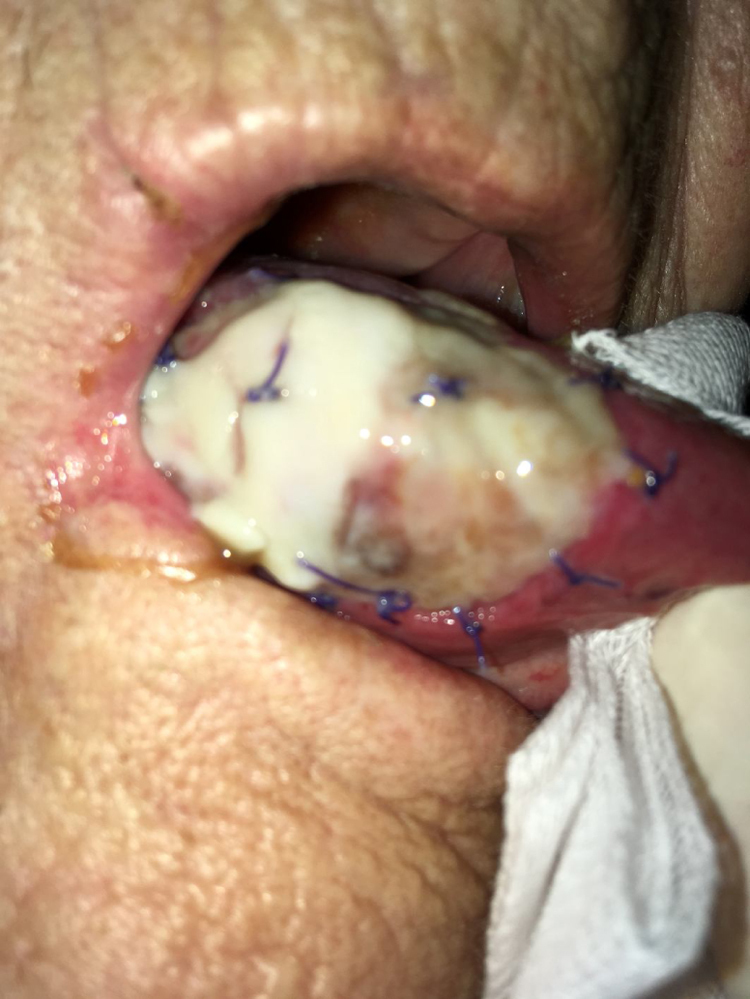
Right tongue 7 days postoperatively: the anterior part of the Tutopatch® starts to be reabsorbed and replaced by the patient’s own mucosa.

**Figure 3:**
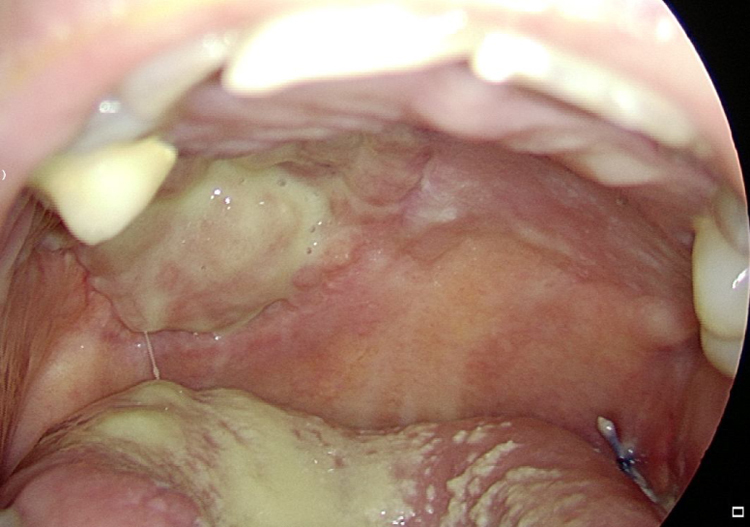
Tutopatch® covers the surgical defect after the resection of a pT1 of the right hard palate. 15 days postoperatively the patient’s own mucosa is progressively replacing Tutopatch®.

**Figure 4:**
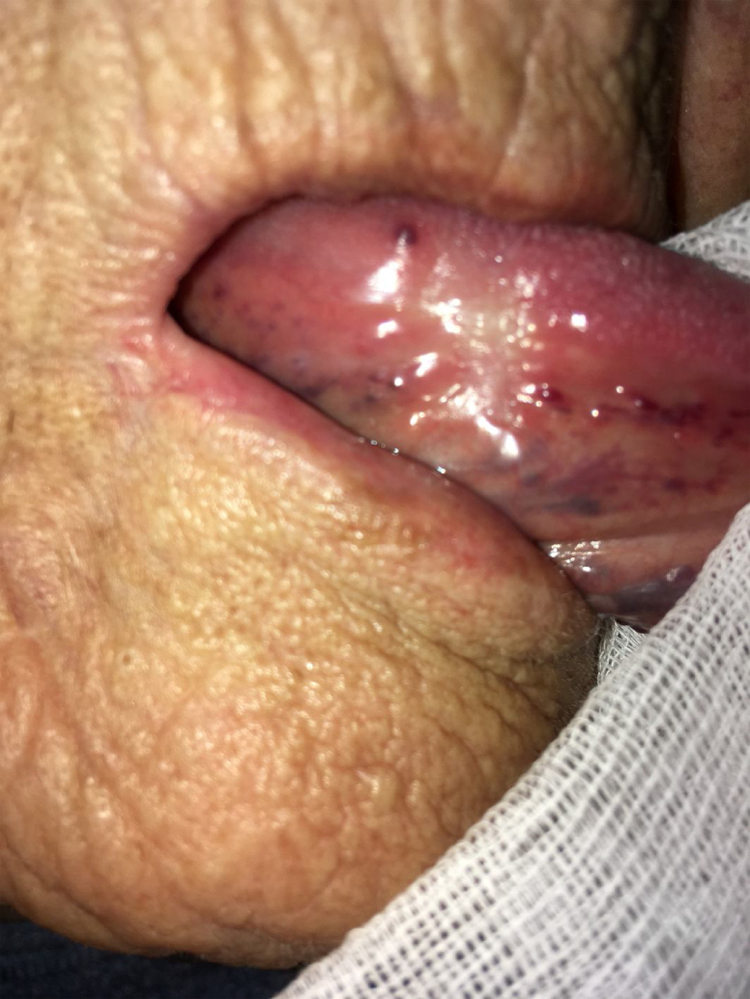
Right tongue 30 days postoperatively: the surgical defect is almost completely healed and Tutopatch® is no longer visible.

**Figure 5:**
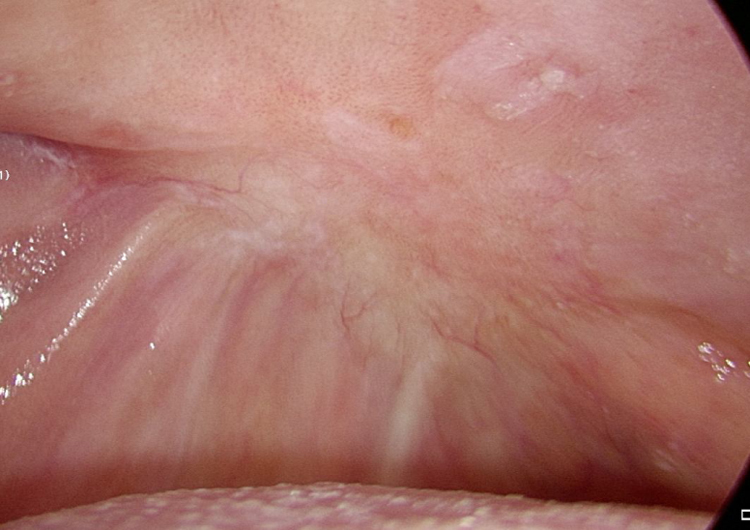
Right hard palate: 60 days postoperatively the surgical defect is completely healed and covered by mucosa.

As stated by the manufacturer, Tutopatch® guides tissue repair and is progressively replaced by new mucosa (Figure 2, Figure 3). In most cases, one month after the reconstruction the graft was no longer visible and the surgical defect appeared completely covered by the patient’s own mucosa (Figure 4, Figure 5). We did not notice differences in healing time in the five patients with diabetes.

The graft was well tolerated by all the patients. Thanks to its thinness and elasticity it did not prevent the recovery of physiologic feeding and speech, as demonstrated by the mean scores reported in the postoperative QoL questionnaire. Moreover the fact that patient’s own mucosa replaces the defect justifies the good taste perceptions reported by our cohort.

In our opinion, the advantages of Tutopatch®, also in comparison with other reconstructive options, are as follows:

• Unlike skin grafts and local flaps it avoids donor site morbidity.
• Unlike skin grafts it has no taking-rate problems because it is progressively reabsorbed.
• Unlike a facial Artery Mucosal Muscular Flap (FAMM) it can also be used in dentate patients with no need for tooth extraction or second surgery for flap autonomization.
• In contrast to classical secondary intention healing after CO_2_ laser resection it covers the surgical defect until the healing process is complete: it prevents wound contact with the saliva and possible resulting infection, it reduces pain, and avoids scar-related tethering.
• It is a faster and easier procedure than skin grafting: it is ready to use and reduces surgical time as no flap has to be harvested. It is particularly important in frail patients who require the shortest possible general anaesthesia time.
• It is progressively absorbed without any need for a second surgery for its removal.
• The process of solvent dehydration allows storage at room temperature: this means there is no need to request it in advance and the surgeon can request the appropriate dimension when he has a precise idea of the size of the defect to be covered.
• Local flaps can be preserved as reconstructive options in the case of local recurrence or secondary tumours.
• It can be used in patients previously treated with local flaps.

The results of this study cannot tell us how Tutopatch® reacts to Radiotherapy (RT) because none of the enrolled patients underwent RT. However, in our opinion it is indicated for covering small surgical defects resulting from the resection of early tumors not requiring adjuvant radiotherapy; moreover the radiation treatment would start 6 weeks after surgery, when Tutopatch® is no longer visible.

## Conclusions

In conclusion, in our opinion Tutopatch® could represent a favorable reconstructive option after surgery for precancerous lesions or early cancer of the oral cavity regardless of the location of the defect. From the present experience it appears to be a fast and easy solution in cases of split-thickness defects. Conversely, advanced tumors are better treated with expanded approaches in which a greater volume of reconstructive tissue is needed, or where a communication between the oral cavity and the neck is present. In such cases, pedicle or free flaps may represent the best choice.

The technique presented does not require new surgical skills, as demonstrated by the fact that the graft was easily placed also by novice surgeons. The cost of Tutopatch® (400Є) could represent a possible drawback, however, overall surgical time and consequently operating room costs can be reduced because the time for local flap or skin graft harvesting is spared.

## Conflicts of interest

The authors declare no conflicts of interest.
